# Amelioration of Melasma Using Quantum Molecular Resonance

**DOI:** 10.1111/jocd.16701

**Published:** 2024-12-08

**Authors:** Eujin Cho, Min‐Hee Kim

**Affiliations:** ^1^ Bethel Dermatology Clinic Seoul Korea; ^2^ University of Hawai'i Cancer Center Honolulu Hawaii USA; ^3^ Division of Endocrinology and Metabolism, Department of Medicine The Catholic University of Korea Seoul Korea

To the Editor,

Treatments for melasma include topical and oral medications, such as Kligmann's compound and topical tranexamic acid, as well as oral options like oral tranexamic acid. However, these treatments may not always be effective, often leading to the search for alternative therapies [1]. Quantum molecular resonance (QMR) is a noninvasive therapy that uses frequency to promote regeneration at the cellular level [2]. No studies have been reported where QMR has been used previously in hyperpigmentation disorders. In this article, we explore the use of QMR in the treatment of melasma in two female patients.

A 38‐year‐old female presented with brownish malar patches and wrinkles. She had a 4‐year history of melasma. She had undergone Nd:YAG laser therapy for five sessions 3 years ago, but reported that she did not notice a significant difference then. The patient was otherwise healthy and consistently employed moisturizer and sunscreen as part of her skincare. She denied any history of pregnancy or the use of oral contraceptive pills. The Melasma Area and Severity Index [3] (MASI) score was 4.8 (Figure 1a). The second patient was a 37‐year‐old female presenting with brownish malar patches for 10 years. She had not tried any treatments before. She was otherwise healthy and consistently used a moisturizer and sunscreen. MASI score was 12 (Figure 1b). The two patients had malar melasma. After informed consent and approval, the two patients underwent QMR (Corage 2.0, Quanteq, Seoul, Korea) for the amelioration of fine wrinkles as well as melasma. The therapy was performed biweekly for five sessions at consistently 35 W per second, a frequency of 4–64 MHz, for 15 min using the ceramic handpiece. The power setting was based on a previous study [4]. The patients reported no discomfort. After five sessions, the patients reported a significant improvement in their melasma lesions. Only frontal photographs, excluding profiles, were taken (MarkVu, PSIPlus, Suwon, Korea). Evaluation of treatment efficacy was done 2 weeks after the fifth treatment session. The first patient had an improved MASI score of 2.4 (Figure 1c). The second patient's MASI score was 9 (Figure 1d). We assessed wrinkle improvement using the Wrinkle Severity Rating Scale [5]. Both patients scored 1 before treatment and the score decreased to 0 after treatment. The patients were satisfied with the results of their melasma and fine wrinkles. Both patients reported 9 for melasma on a scale of 1 (least satisfactory) to 10 (most satisfactory). For fine wrinkles, Patient 1 reported 10, and Patient 2 reported 9, respectively. No adverse effects were noted during or after therapy. No recurrence was noted during the 2 weeks of follow‐ups after the cessation of treatment.

**FIGURE 1:**
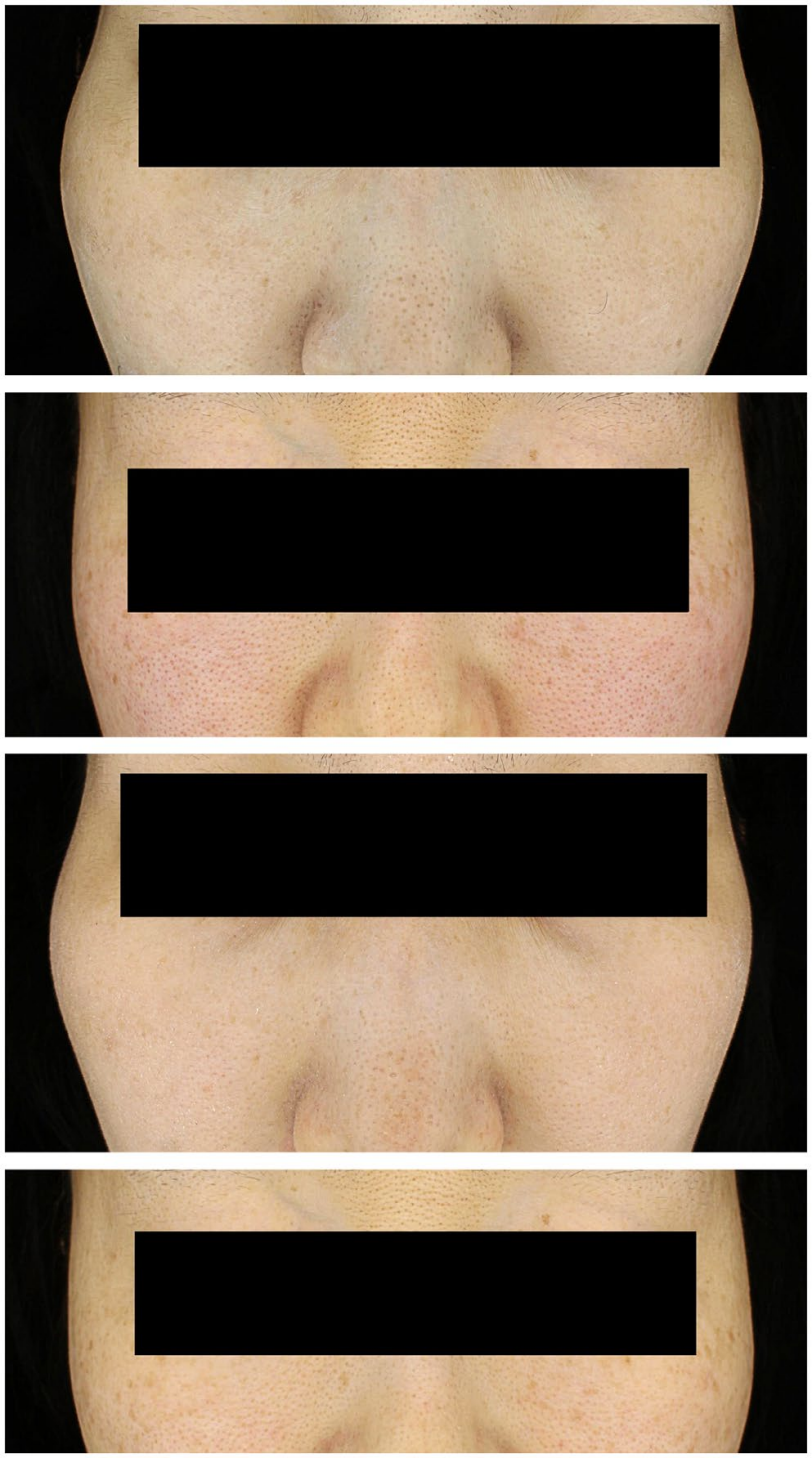
(a) Baseline photo of Patient 1, MASI score of 4.8. (b) Baseline photo of Patient 2, MASI score of 12. (c) Patient 1 after treatment, MASI score of 2.4. (d) Patient 2 after treatment, MASI score of 9.

The use of QMR in the treatment of melasma is a new concept. QMR aims to promote healing by using specific frequencies to stimulate cellular activity [2]. QMR therapy has been used to treat various medical conditions, including inflammation and wrinkles, and has shown promising results [4, 6]. No specific adverse effects have been identified for QMR [2, 4, 6].

Novel agents for melasma target multiple pathophysiological mechanisms, one of which may be the overproduction of reactive oxygen species and the associated inflammatory response. Preliminary evidence suggests that QMR exhibits anti‐inflammatory properties, possibly by generating radiofrequency resonance waves that influence molecular bonding and promote cellular regeneration [3]. The effectiveness of QMR in reducing edema after total knee arthroplasty in a clinical trial has been reported [6]. The study indicates a decrease in inflammatory cells, potentially accelerating healing [6]. QMR has demonstrated clinical applications in reducing skin laxity [4], with speculations of anti‐inflammation, which could contribute to observed enhancements. QMR might contribute to reducing the inflammatory component of melasma. By targeting and modulating the underlying inflammatory pathways, QMR could potentially help alleviate the symptoms associated with this condition.

While our findings are encouraging, we recognize limitations within our study. Notably, the absence of a control group and the lack of comparison with other energy‐based devices limit the strength of our conclusions. Studies have utilized energy‐based devices for melasma, assessing treatment responses and histological changes to gauge efficacy. Studies employing intense pulsed light or fractional lasers have provided insights into the potential mechanisms of action, such as melanin dispersion and reduction in melanocyte activity [1].

In our cases, QMR was well tolerated, and both patients reported significant improvement in their symptoms after 12 weeks of treatment. While further studies are needed to confirm the efficacy of QMR in the treatment of melasma as well as to explore its mechanism of action. QMR may be a promising alternative treatment option for patients with melasma. While this theory is speculative and based on a conceptual understanding of QMR, it can provide a direction for future research.

## Conflicts of Interest

The authors declare no conflicts of interest.

## Data Availability

The data that support the findings of this study are available from the corresponding author upon reasonable request.
